# Succinic semialdehyde dehydrogenase deficiency: a metabolic and genomic approach to diagnosis

**DOI:** 10.3389/fgene.2024.1405468

**Published:** 2024-06-19

**Authors:** Kevin E. Glinton, Charul Gijavanekar, Abbhirami Rajagopal, Laura P. Mackay, Kirt A. Martin, Phillip L. Pearl, K. Michael Gibson, Theresa A. Wilson, V. Reid Sutton, Sarah H. Elsea

**Affiliations:** ^1^ Department of Molecular and Human Genetics, Baylor College of Medicine, Houston, TX, United States; ^2^ NeoGenomics Laboratories, Aliso Viejo, CA, United States; ^3^ Boston Children’s Hospital, Harvard Medical School, Boston, MA, United States; ^4^ Department of Pharmacotherapy, College of Pharmacy and Pharmaceutical Sciences, Washington State University, Spokane, WA, United States; ^5^ Baylor Genetics Laboratories, Houston, TX, United States

**Keywords:** SSADHD (succinic semialdehyde dehydrogenase deficiency), succinic semialdehyde dehydrogenase, GABA catabolism, ALDH5A1, GHB (4-hydroxybutyric acid), 2-pyrrolidinone, GABA-T (GABA transaminase)

## Abstract

Genomic sequencing offers an untargeted, data-driven approach to genetic diagnosis; however, variants of uncertain significance often hinder the diagnostic process. The discovery of rare genomic variants without previously known functional evidence of pathogenicity often results in variants being overlooked as potentially causative, particularly in individuals with undifferentiated phenotypes. Consequently, many neurometabolic conditions, including those in the GABA (gamma-aminobutyric acid) catabolism pathway, are underdiagnosed. Succinic semialdehyde dehydrogenase deficiency (SSADHD, OMIM #271980) is a neurometabolic disorder in the GABA catabolism pathway. The disorder is due to bi-allelic pathogenic variants in *ALDH5A1* and is usually characterized by moderate-to-severe developmental delays, hypotonia, intellectual disability, ataxia, seizures, hyperkinetic behavior, aggression, psychiatric disorders, and sleep disturbances. In this study, we utilized an integrated approach to diagnosis of SSADHD by examining molecular, clinical, and metabolomic data from a single large commercial laboratory. Our analysis led to the identification of 16 patients with likely SSADHD along with three novel variants. We also showed that patients with this disorder have a clear metabolomic signature that, along with molecular and clinical findings, may allow for more rapid and efficient diagnosis. We further surveyed all available pathogenic/likely pathogenic variants and used this information to estimate the global prevalence of this disease. Taken together, our comprehensive analysis allows for a global approach to the diagnosis of SSADHD and provides a pathway to improved diagnosis and potential incorporation into newborn screening programs. Furthermore, early diagnosis facilitates referral to genetic counseling, family support, and access to targeted treatments–taken together, these provide the best outcomes for individuals living with either GABA-TD or SSADHD, as well as other rare conditions.

## 1 Introduction

So-called “rare” and “ultra-rare” disorders are estimated to affect between 1.5% and 6.2% of the global population (Chiu et al., 2018; Ferreira, 2019; Nguengang Wakap et al., 2020). Inborn errors of metabolism are an important subset of these disorders and themselves are estimated to occur in approximately 50.9 per 100,000 live births worldwide (Waters et al., 2018). Despite advances in newborn screening and next-generation sequencing, however, many rare or ultra-rare metabolic disorders remain difficult to diagnose in a timely, and non-invasive manner. This also limits our ability to gain insight into mechanisms fundamental to more common diseases along with limiting the development of much-needed, targeted therapies (Kaufmann et al., 2018; Tabor and Goldenberg, 2018). Because of this, efforts are being made to integrate available “omic” techniques in the diagnosis of rare/ultra-rare disorders as a means of shortening the diagnostic odyssey and better delineating the phenotypic and genotypic spectrum of disease (Crowther et al., 2018; Ahmed, 2020; Kerr et al., 2020; Stenton et al., 2020; Papandreou et al., 2022). Succinic semialdehyde dehydrogenase deficiency (SSADHD, OMIM #271980) due to biallelic variants in *ALDH5A1*, is one such rare neurometabolic disorder (Jakobs et al., 1981; Gibson et al., 1983). While the actual incidence and prevalence of the disease are currently unknown, at least 450 individuals have been described worldwide (Pearl et al., 2003a; Pearl et al., 2019; Pearl et al., 2021). There are, however, numerous challenges in the diagnosis and treatment of this disease including its rarity and limited knowledge of the mutational spectrum and phenotypic variability.

Here, we briefly review the diagnosis and clinical features of individuals with SSADHD. We also describe our efforts to curate and characterize the likely pathogenic or pathogenic *ALDH5A1* variants reported in a large diagnostic laboratory to uncover previously unrecognized cases of SSADHD. We use these data along with reports from the literature and other available molecular databases to estimate the prevalence of this rare disease in the general population. Furthermore, we describe the metabolomic biomarker profiles generated from biofluids obtained from individuals with SSADHD and use these data along with available clinical and molecular information to illustrate the utility of a comprehensive, integrated approach to diagnosis. In this way, we aim to propose a more useful approach for broad population screening for SSADHD which would thereby lead to a more efficient and equitable approach to diagnosis.

## 2 Features of SSADH deficiency

### 2.1 Biochemistry

**FIGURE 1:**
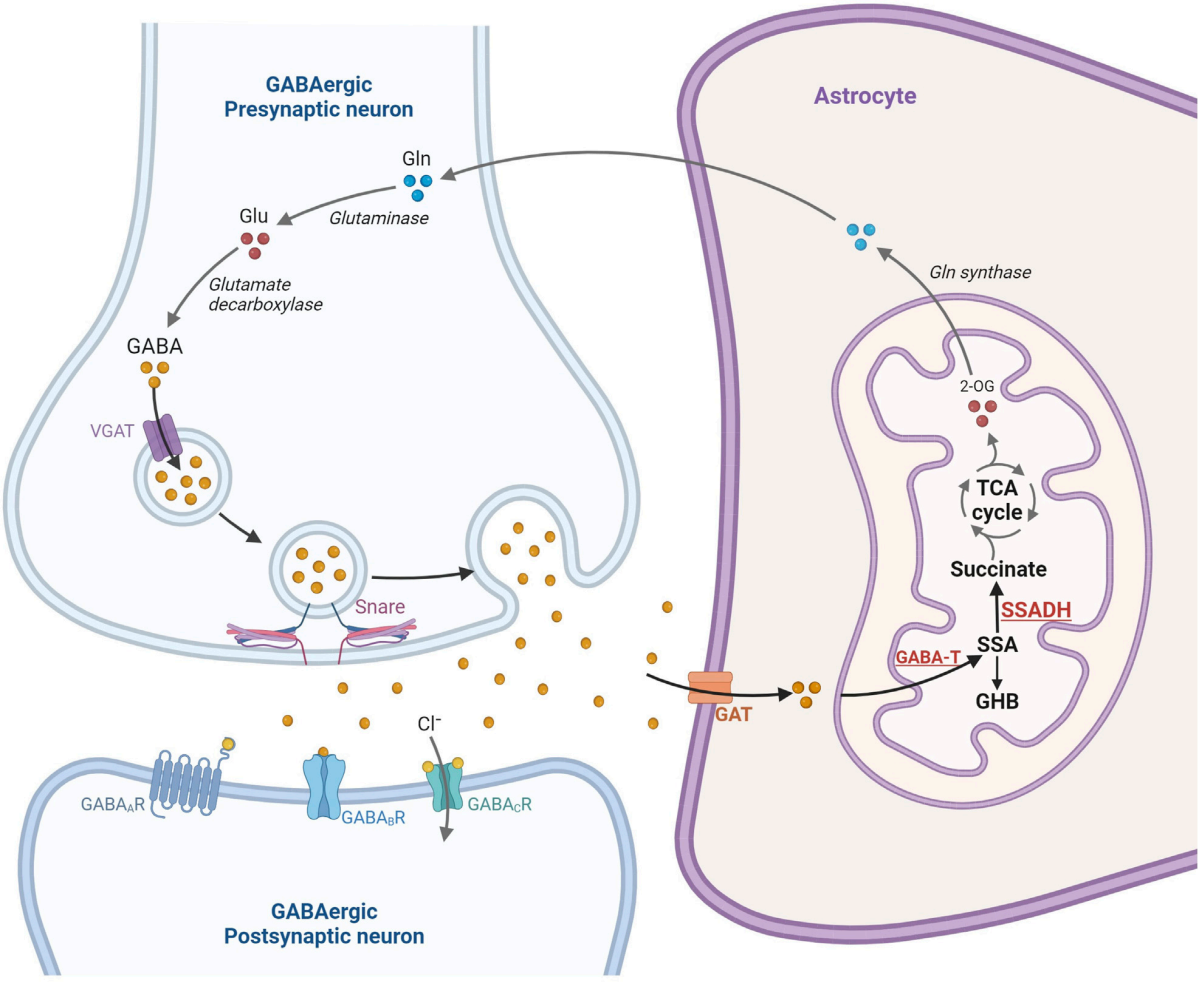
Overview of the gamma-aminobutyric acid (GABA) release, neurotransmission in the GABAergic-synapse and catabolism in the astrocyte mitochondria. Glutamate is one of the main sources of GABA formation via glutamic acid decarboxylase (GAD1). In response to electrophysiological activation, GABA is released into the presynaptic cleft where it binds three receptors, GABA_A_, GABA_B_, and GABA_C_ receptors. GABA is subsequently removed from the synaptic cleft by uptake in the astrocytes and catabolized in the astrocyte mitochondria to form succinic semialdehyde (SSA), gamma-hydroxybutyrate (GHB) and succinate. Succinate is subsequently channeled into the TCA cycle (Prepared using BioRender.com).

Succinic semialdehyde dehydrogenase is the final of two enzymatic steps in the catabolism of gamma-aminobutyric acid (GABA) (Pearl et al., 2019). An NAD + -dependent enzyme, SSADH converts succinic semialdehyde to succinic acid prior to its entry into the citric acid cycle (Figure 1). Loss of enzyme activity leads to an excess of succinic semialdehyde which itself may be converted to gamma-hydroxybutyric acid (GHB or 4-hydroxybutyric acid) by the enzyme 4-hydroxybutyrate dehydrogenase. Significant elevations in GHB have been observed in the blood, urine, and cerebrospinal fluid of affected individuals, which is how patients were initially diagnosed with the disorder (Gibson et al., 1990; Struys et al., 2005). However, some reports describe individuals with molecularly and enzymatically confirmed SSADHD and normal or only mildly elevated GHB excretion, as well as elevated GHB in individuals with reportedly normal enzyme activity (Bonham et al., 1994; Pitt et al., 1997; Gibson et al., 1998). A recent study also highlighted that while the level of GHB decreases with age in healthy individuals, the same effect may also be observed in affected individuals (Brown et al., 2019; Brown et al., 2020). Given these findings, GHB may not always serve as an accurate biomarker, thereby necessitating further genetic or enzymatic analysis in cases strongly suspected of having SSADHD. Additionally, while SSADH enzyme assays have historically been utilized in research studies, such testing is not currently widely available for clinical diagnosis (Gibson et al., 1991; Gibson et al., 1994; Hogema et al., 2001).

### 2.2 Mechanism of Disease

While the exact patho-mechanism of SSADHD remains unclear, it is commonly assumed that most neurologic symptoms are due to elevations in GABA due to its role as the primary inhibitory neurotransmitter via GABA_A_ and GABA_B_ receptors (Reis et al., 2012; Tang et al., 2021). GABA signaling is also known to be critical for developmental neuronal migration and synaptogenesis, as well as playing an important role in neuronal stem cell proliferation, which may explain some of the early onset developmental delays in patients with SSADHD (Wang and Kriegstein, 2009; Pontes et al., 2013). As a GABA metabolite, GHB is thought to act as a neuromodulator at endogenous concentrations and has also been shown to modify the release of other important neurotransmitters like GABA, glutamate, and dopamine (Maitre et al., 2016; Felmlee et al., 2021). GHB exerts its effects by binding to both its own unique receptor (SLC52A2, also called GHBh1), as well as GABA_B_ receptors (Andriamampandry et al., 2007; Yao et al., 2010; Absalom et al., 2012; Bay et al., 2014; Maitre et al., 2016) and at pharmacologic doses, is known to cause euphoria, drowsiness, and a loss of inhibition. Because of these effects, GHB has been exploited both as a treatment for conditions like narcolepsy and as a drug of abuse, through its effects on dopamine as mediated by its GABA_B_ receptor binding (White, 2017; Felmlee et al., 2021). Less clear, however, is what role the molecule may play in terms of neuronal development and why individuals with SSADHD do not exhibit the same symptoms as those taking the drug illicitly.

### 2.3 Disease Phenotype

Clinically, patients with SSADHD usually present within the first few years of life with developmental delays, particularly with delayed, limited, or deficient speech acquisition (Pearl et al., 2003b; Pearl et al., 2009). Other early symptoms may include hyporeflexia, ataxia, autism spectrum disorders, sleep disorders, and behavioral disturbances (including anxiety, obsessive-compulsive disorder, inattention/hyperactivity and sometimes aggression) (Pearl et al., 2003b; Gibson et al., 2003; Knerr et al., 2008; Pearl et al., 2009). Medical histories may also reveal early features like neonatal hypotonia, poor feeding, and respiratory distress. Seizures may develop in up to 50% of affected patients, with increasing prevalence with age (Pearl et al., 2011; DiBacco et al., 2019; Pearl et al., 2021). These seizures may be characterized by a variety of semiologies, including generalized tonic-clonic, absence, focal, and in some cases, myoclonic seizures (Pearl et al., 2011). Electroencephalographic (EEG) findings include background abnormalities/slowing, spike discharges, electrographic status epilepticus during slow wave sleep, and photosensitivity (Pearl et al., 2011). Magnetic resonance imaging studies in affected patients have identified increased T2-weighted signal in the globus pallidus, subcortical white matter, subthalamic nuclei, cerebellar dentate nuclei, and brainstem, as well as cerebral and cerebellar atrophy, and delayed myelination (Yalcinkaya et al., 2000; Ziyeh et al., 2002; Afacan et al., 2021). Metabolic strokes have also been rarely described in the setting of acute illnesses (Wang et al., 2016; Yoganathan et al., 2020; Yoganathan et al., 2021). Magnetic resonance spectroscopy studies have identified increased GABA concentrations, as well as elevations of GHB and homocarnosine (Al-Essa et al., 2000; Ethofer et al., 2004; Afacan et al., 2021).

### 2.4 Treatment and Therapies

Currently, there is no specific treatment for SSADHD, and most therapies focus on management of symptoms. Seizures, for instance, are treated with standard therapies, though valproate is usually avoided due to its inhibition of residual SSADH enzyme activity (Shinka et al., 2003). Vigabatrin therapy has been trialed extensively due to its known role as an inhibitor of GABA transaminase (Tolman and Faulkner, 2009). While the drug indeed does lead to an increase in GABA and noncommittal decrease in GHB, clinical response to this therapy has been largely mixed with some milder patients exhibiting a positive response while others experience little to no change in symptoms (Gibson et al., 1989; Gibson et al., 1995; Matern et al., 1996; Gropman, 2003; Leuzzi et al., 2007; Vogel et al., 2013). Attempts to improve upon the effects of vigabatrin led to the discovery and development of (3-aminopropyl) (n-butyl)phosphinic acid (known as SGS-742), another GABA_B_ receptor antagonist. Following pilot studies in mouse models, a human randomized control of the drug was conducted, and though the drug did not cause an increase in adverse events, there was no discernible improvement in cognition or cortical excitability (Schreiber et al., 2021). The amino acid taurine has also been trialed as a potential treatment for SSADHD based on its postulated role as an alternative substrate for the GABA transporter and its abilities to modulate ER-stress (Pearl et al., 2014; Jakaria et al., 2019). In an open-label study, however, there were no statistically significant improvements observed in adaptive behavior, no changes in cerebrospinal fluid (CSF) GABA concentrations, and paradoxically increased cortical excitability on paired-pulse transcranial magnetic stimulation (Pearl et al., 2014; Schreiber et al., 2016). Attempts to develop newer and more efficient therapies continue to this day along with the optimization of supportive therapies (Nylen et al., 2008; Pearl et al., 2009; Vogel et al., 2013; Vogel et al., 2018).

## 3 Methods

### 3.1 Subjects and Biospecimens

Data from all referenced cases (1 through 16, also referred to as BG-BCM cohort) presented here were derived from studies conducted under clinical diagnostic testing at Baylor Genetics Laboratories (Houston, TX, United States) and Baylor College of Medicine (BCM) Institutional Review Board (IRB) approved protocols H-32701 and H-35388.

EDTA plasma and urine samples from subjects 17 through 24 were obtained from the Succinic Semialdehyde Dehydrogenase (SSADH) Deficiency Biorepository, Rosamund Stone Zander Translational Neuroscience Center, Boston Children’s Hospital (Boston, MA, United States). This study was approved by the Boston Children’s Hospital Institutional Review Board approved protocol IRB 09-02-0043.

Molecular testing for the remaining cases in the BG-BCM cohort included either full gene Sanger sequencing, or exome sequencing performed at Baylor Genetics Laboratories. Molecular testing for cases 11 and 13 was performed at MNG Laboratories (Atlanta, GA, United States).

### 3.2 Sample storage

EDTA-plasma or urine samples were stored in −80 deg C freezer. Pre-analytically, upon collection of blood sample in an EDTA tube, plasma was separated and frozen, and remained frozen until testing. Thawed samples were not accepted for testing. Plasma with evidence of hemolysis was not accepted for testing. Similarly, urine samples were only accepted when received frozen. Samples were kept on ice through the pre-analytical phase of sample handling, processing, and extraction.

### 3.3 Metabolomic Analyses

**FIGURE 2:**
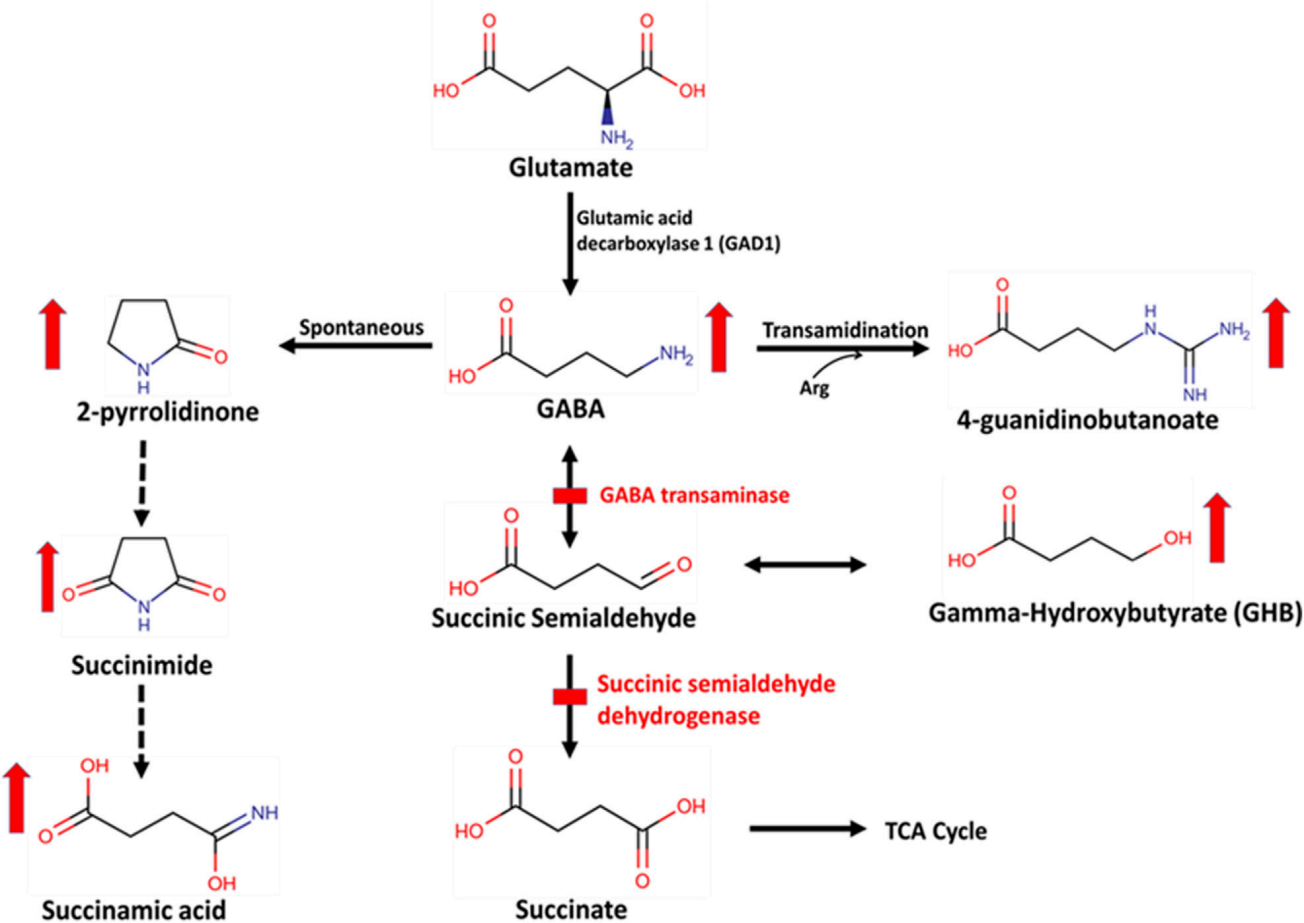
Biochemical pathway of GABA metabolism by GABA transaminase (GABA-T) and succinic semialdehyde dehydrogenase (SSADH). This is a two-step enzymatic pathway. In step 1, GABA is transaminated to form succinic semialdehyde by GABA-T. GABA can also be spontaneously converted to a stable lactam ring form, 2-pyrrolidinone, which is then converted to succinimide and succinamic acid or into 4-guanidinobutanoate via a transamidination reaction with arginine (Arg). In step 2, succinic semialdehyde is oxidized to form succinate by SSADH. Succinic semialdehyde may then be converted to gamma-hydroxybutyrate (GHB).

Clinical untargeted metabolomic profiles were generated from EDTA plasma derived from venous whole blood and urine obtained from five subjects–case numbers 2, 11, 13, 14, and 15 in the BG-BCM Cohort. To further validate metabolomic findings from the BG-BCM cohort, we acquired additional plasma and urine samples from known individuals with SSADHD (cases 17 through 24) from the SSADH Biobank. Urine samples were normalized per creatinine levels. Metabolomic profiling was performed at Baylor Genetics (Houston, TX, United States) and Metabolon, Inc. (Morrisville, NC, United States) using liquid chromatography mass spectrometry (LC/MS) analysis as previously described (Miller et al., 2015; Glinton et al., 2018; Alaimo et al., 2020; Ford et al., 2020; Liu et al., 2021). Small molecules were extracted from 100 µL of sample in an 80% methanol solution, and samples were run on four independent platforms, each able to measure a different set of metabolites: (1) positive ionization with Waters BEH C18 chromatographic separation of hydrophilic compounds (LC/MS/MS Pos Polar), (2) positive ionization with Waters BEH C18 chromatographic separation of hydrophobic compounds (LC/MS/MS Pos Lipid), (3) negative ionization with Waters BEH C18 optimized conditions (LC/MS/MS Neg), and (4) negative ionization with Waters BEH Amide (HILIC) chromatography (LC/MS/MS Polar). All chromatography was performed using a Waters Acquity UPLC (Waters, Milford, MA) held at 40°C–50 °C. Deuterated internal standards, including 2-pyrrolidinone were used to monitor performance and serve as retention index markers and chosen based on their broad chemical structures, biological variety, and their elution spectrum on each of the arms of the platform. The chemical structures of known metabolites were identified by matching the ions’ chromatographic retention index, nominal mass, and mass spectral fragmentation signatures with reference library entries created from authentic standard metabolites under the identical analytical procedure as the experimental samples. Semiquantitative analysis of metabolites in each sample was achieved by comparing the individual patient sample to a set of invariant anchor specimens included in each batch. Raw spectral intensity values were then normalized to the anchor samples, log transformed, and compared to a normal reference population to generate Z-scores. Results were considered abnormal if the z-score for a compound was equal to or greater than two standard deviations above or below the mean of the control reference population (*n* = 395). The metabolomic signature of SSADHD was identified based on characteristic perturbations in the concentrations of GABA metabolites and precursors including 2-pyrrolidinone, 4-guanidinobutanoate, succinamic acid, and succinimide (Figure 2) (Kennedy et al., 2019). Simple linear regression analysis of metabolite z-scores was performed to assess their association with age of the affected individuals. The average metabolite z-score of pediatric and adult age-groups were compared statistically using a two-tailed Student’s t-test. Complete metabolomic profiles are provided in Supplementary Table S5 for each case.

### 3.4 *ALDH5A1* population variant assessment and disease prevalence estimates

*ALDH5A1* pathogenic variants were collated from the historical literature, ClinVar (https://www.ncbi.nlm.nih.gov/clinvar/), and an internal SSADH Deficiency Association database (https://www.ssadh.net) to provide the most complete assessment of disease-causing variants in *ALDH5A1*. Variants were excluded if they could not be confirmed and validated in the Allele Registry (http://reg.clinicalgenome.org/redmine/projects/registry/genboree_registry/landing) or in other human genome reference databases, including ClinVar (https://www.ncbi.nlm.nih.gov/clinvar/) and ClinVar Miner (https://clinvarminer.genetics.utah.edu). The Allele Registry is a genome integration database supported by the ClinGen Project (https://clinicalgenome.org). Nomenclature for all variants follows HGVS standards.

Population-based exome and whole genome data from the Genome Aggregation Database (gnomAD) were interrogated for *ALDH5A1* pathogenic/likely pathogenic variants reported as disease-causing variants. All *ALDH5A1* variants listed in gnomAD v4.0.0 (https://gnomad.broadinstitute.org) were curated to determine predicted pathogenicity. Two primary transcripts for *ALDH5A1* are reported: (1) the canonical transcript, *ALDH5A1* NM_17040, which contains 11 exons, and (2) *ALDH5A1* NM_001080, which contains 10 exons, is the MANE Select transcript, and is represented in gnomAD v4.0.0. The transcript NM_17040 differs from NM_001080 by the presence of an additional in-frame exon with 13 amino acids in the middle of the protein. Differences in function and expression of these two transcripts are not described.

Protein truncating variants (including frameshifting variants, stop-gains, start-loss, splice site variants at +/-1 and +/-2 location) which are present in gnomAD but not yet reported to be associated with SSADHD in literature or ClinVar database were classified as likely pathogenic. All known pathogenic or likely pathogenic variants that have been reported to be associated with SSADHD either in ClinVar database, published patient data, confirmed variants from our cohort, and the SSADHD Biobank were also identified. Taken together, these novel and known likely pathogenic or pathogenic variants in the general population represented in gnomAD v4.0.0 were used to calculate carrier frequency and to estimate disease prevalence in this population. Additional measures of carrier frequency and disease prevalence were performed by applying stringent computational predictions of variant deleteriousness with a CADD score cut-off value of ≥28.0 for nonsynonymous variants and SpliceAI score (≥0.2) for potentially spliceogenic variants.

Carrier frequency was assessed by determining the minor allele frequency of all pathogenic/likely pathogenic variants across all populations present in the publicly accessible gnomAD database v4.0.0 (https://gnomad.broadinstitute.org/). The minor allele frequency (MAF) of each variant was used to determine overall carrier frequency and disease prevalence, as described previously (Cappuccio et al., 2019; Elsea et al., 2020; Martin et al., 2021). Disease prevalence estimates were determined based on cumulative *ALDH5A1* pathogenic/likely pathogenic allele frequency within this database. Data were assumed to be in Hardy-Weinberg equilibrium, and standard Hardy-Weinberg calculations were used to determine carrier frequency and disease.

### 3.5 Estimated disease frequency in a clinically referred population

During the period 2014–2022, we assessed 5,000 plasma, urine, and CSF untargeted metabolomic samples at our laboratory. During the same period, we assessed a total of 17,000 Sanger, exome, or genome sequencing samples. Collectively, we considered these samples as our clinically referred population of 22,000 samples. Among these, we assessed the number of individuals confirmed to be diagnosed with SSADHD and GABAT-D to determine the estimated disease frequency in a clinically referred population.

## 4 Results

### 4.1 SSADHD Cohort Analysis

We identified 16 individuals in the BG-BCM cohort (Cases 1–16, Table 1) with possible or known SSADHD. In addition, we also acquired plasma and urine samples from eight unrelated individuals from the Boston Children’s Hospital SSADHD Biobank (Cases 17–24, Table 1). Of the 24 individuals, 10 (41.6%) were males and 14 (58.3%) were females. At the time of genetic testing or Biobank sample collection and analysis, participant ages ranged from 6 months to 44 years, with a mean age of 11.5 ± 12.66 years and median age 7 years. Among these, 18 individuals were in the pediatric age-group ranging from 6 months to 12 years and six individuals were in the adult age-group ranging from 22 years to 44 years. Among the BG-BCM cohort, the phenotypic presentation or most common reason for referral included developmental delays and hypotonia, while ataxia, seizures, abnormal brain MRI, autistic features, failure to thrive, or stroke were indicated in others (Table 1).

**TABLE 1:** Demographics, molecular genotype, clinical characteristics, and laboratory findings of patients with SSADHD in our cohort.

BG-BCM cohort							
Case #	Age (y)	Sex	Ethnicity	Clinical symptoms	UOA or urine GHB (mmol/molCr)*	Allele 1 ALDH5A1:NM_001080.3 (NP_001071.1)	Allele 2 ALDH5A1:NM_001080.3 (NP_001071.1)
1	0.5	M	Middle Eastern	Developmental delays, hypotonia, elevated GHB in urine	Elevated GHB	c.668G>A (p.Cys223Tyr)	c.668G>A (p.Cys223Tyr)
2	0.5	M	Not provided	Developmental delays, hypotonia, failure to thrive	Elevated GHB	c.1597G>A (p.Gly533Arg)	c.1015–2A>C
3	1	F	South Asian	Developmental delays, hypotonia	Not available	c.768_784del (p.Ile257GlufsTer12)	c.768_784del (p.Ile257GlufsTer12)
4	1	F	Not provided	Developmental delays, hypotonia, unsteady gait, increased T2 signal bilateral globus pallidi	Elevated GHB	c.649G>A (p.Ala217Thr)	c.649G>A (p.Ala217Thr)
5	1	F	Hispanic American	Developmental delays, hypotonia	“Abnormal”	c.379_380del (p.Trp127ValfsTer8)	c.379_380del (p.Trp127ValfsTer8)
6	1	F	Not provided	Developmental delays, hypotonia, abnormal MRI, abnormal urine organic acids	“Abnormal"	c.1226G>A (p.Gly409Asp)	c.1323dup (p.Pro442AlafsTer19)
7	2	M	Asian	Seizures, microcephaly, hypotonia, global developmental delays	Not available	c.1501_1503del (p.Glu501del)	c.1501_1503del (p.Glu501del)
8	4	F	European	Severe speech delay, mixed developmental delays, abnormal urine organic acids	“Abnormal”	c.967_968dup (p.Gln323HisfsTer4)	c.1597G>A (p.Gly533Arg)
9	5	M	Hispanic	Significant developmental delays, ataxia, cerebellar atrophy, hypotonia, absent speech	Not available	c.111_122delinsG (p.Ala38GlyfsTer94)	c.608C>T (p.Pro203Leu)
10	7	F	Not provided	Globus pallidus stroke, elevated GHB in urine	Elevated GHB	c.416C>A (p.Ala139Asp)	c.1015–2A>C
11	10	M^a^	European	Developmental delays, hypotonia, non-verbal	Elevated GHB	c.612G>A (p.Trp204Ter)	c.1234C>T (p.Arg412Ter)
12	11	F	African American	Developmental delays, elevated GHB in urine	Elevated GHB	c.380G>A (p.Trp127Ter)	c.431C>A (p.Ala144Asp)
13	12	F^a^	European	Developmental delays, hypotonia	Elevated GHB	c.612G>A (p.Trp204Ter)	c.1234C>T (p.Arg412Ter)
14	22	F^b^	European	Known SSADHD, details not provided	Not available	c.803G>A (p.Gly268Glu)	c.1558G>C (p.Gly520Arg)
15	25	F^b^	European	Known SSADHD, details not provided	Not available	c.803G>A (p.Gly268Glu)	c.1558G>C (p.Gly520Arg)
16	44	M	European	Developmental delays, hypotonia, seizures, autistic features, weakness, unsteady gait	Not available	c.1597G>A (p.Gly533Arg)	c.1234C>T (p.Arg412Ter)
SSADHD Biobank							
17	2	F		Known SSADHD	4,323.0	c.612G>A (p.Trp204Ter)	c.1597G>A (p.Gly533Arg)
18	5	M		Known SSADHD	431.4	c.104_127del (p.Ser35Ter)	c.1015–2A>C
19	7	F		Known SSADHD	228.0	c.612G>A (p.Trp204Ter)	c.803G>A (p.Gly268Glu)
20	10	F		Known SSADHD	302.0	c.608C>T (p.Pro203Leu)	c.608C>T (p.Pro203Leu)
21	11	F		Known SSADHD	1,431.0	c.1226G>A (p.Gly409Asp)	c.1323dup (p.Pro442AlafsTer18)
22	25	M		Known SSADHD	74.5	c.754G>T (p.Gly252Cys)	c.754G>T (p.Gly252Cys)
23	27	M		Known SSADHD	81.2	c.612G>A (p.Trp204Ter)	c.612G>A (p.Trp204Ter)
24	41	M		Known SSADHD	60.8	c.612G>A (p.Trp204Ter)	c.1015–2A>C

^a^ Full Siblings

^b^ Full Siblings

Age at sample collection

* Values provided on test requisition or per clinical report. “Abnormal” or “Elevated GHB” as indicated in the requisition form - not tested at Baylor Genetics.

GHB: gamma-hydroxybutyrate (4-hydroxybutyrate).

GHB, normal value: 0–7 mmol/mol creatinine.

The individuals in the BG-BCM Cohort (Cases 1–16) were identified by surveying over 17,000 clinical exome sequencing and Sanger sequencing databases for cases carrying biallelic, pathogenic, or likely pathogenic variants in *ALDH5A1* at Baylor Genetics. Novel variants were curated based on current ACMG/AMP variant interpretation criteria (Richards et al., 2015). This comprehensive review of sequencing data identified 14 individuals with biallelic variants in *ALDH5A1* (Table 1)*.* In two individuals (Cases 11 and 13), molecular sequencing was carried out at another laboratory with sequencing information provided to aid in the interpretation of metabolomic analysis. In these instances, the finding of biallelic pathogenic or likely pathogenic variants in *ALDH5A1* in the setting of characteristic clinical features and/or elevated levels of GHB were consistent with the diagnosis of SSADHD. The clinical metabolomics database of the laboratory was also surveyed for cases with a significant pattern of perturbations in GABA metabolism analytes. When available, corresponding molecular and metabolomic data were combined with provided clinical information to confirm or rule out SSADHD as a clinical diagnosis. SSADHD Biobank samples (Cases 17–24) were from patients with previously confirmed SSADHD and are shown with *ALDH5A1* genotypes and urine GHB level at the time of evaluation (Table 1).

**FIGURE 3:**
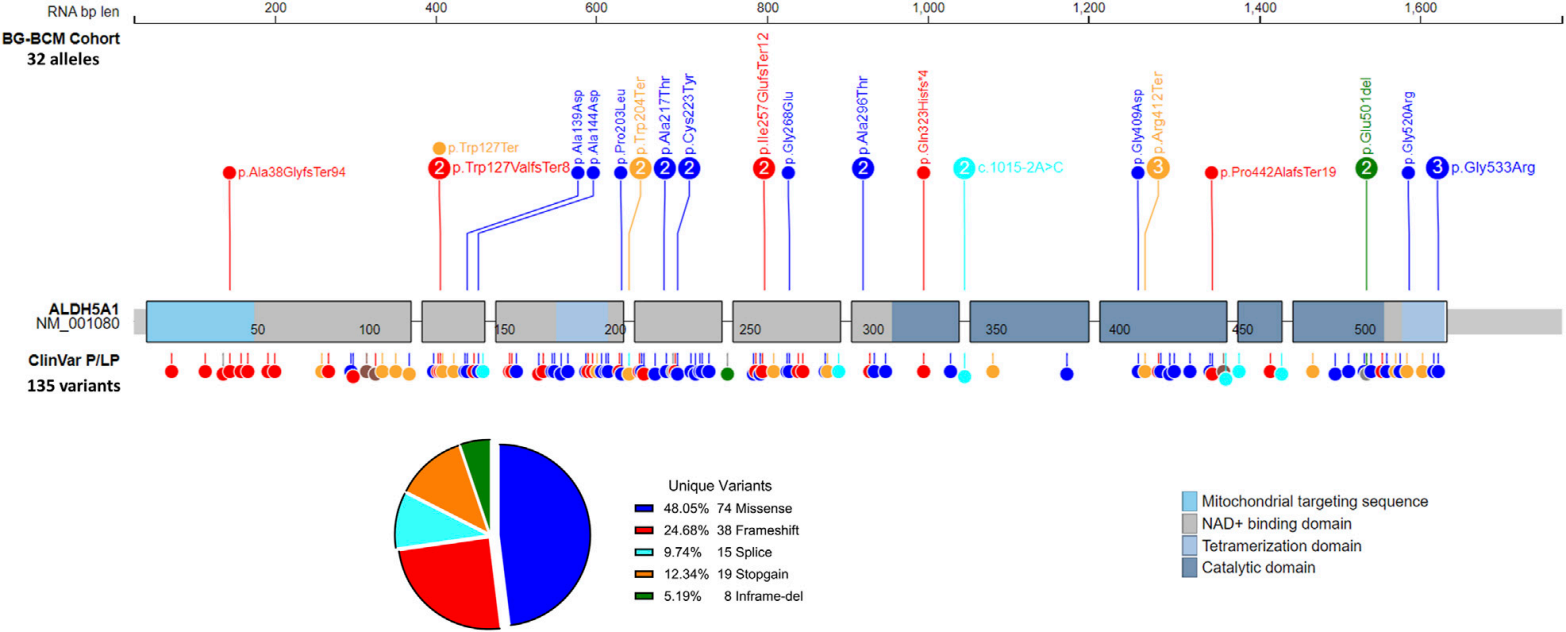
Summary of variants identified in SSADHD patients. All pathogenic or likely pathogenic variants detected in SSADHD patients in the BG/BCM cohort are depicted and labeled with variant frequency within the cohort. ClinVar pathogenic and likely pathogenic variants are also depicted.

Among the 16 identified cases in the BG-BCM cohort, four individuals harbored homozygous pathogenic or likely pathogenic variants. A total of 19 unique variants were identified in our study, 3 (15.7%) of which are novel. All three variants were classified as pathogenic [c.111_122delinsG (p.Ala38GlyfsTer94); c.768_784del (p.Ile257GlufsTer12); and c.380G>A (p.Trp127Ter)] given that they are predicted to lead to premature translation termination (Supplementary Table S3). A summary of known disease associated variants in SSADHD in BG-BCM cohort and those reported in ClinVar database have been shown for an overview (Figure 3).

### 4.2 Analysis of *ALDH5A1* variants reveals population-specific prevalence estimates

*ALDH5A1* variants were considered pathogenic (P) or likely pathogenic (LP) if the nucleotide change (1) resulted in a premature termination codon (stop-gain), (2) occurred at a splice site (within +/- 0–2 bp from reported, canonical splice site), (3) resulted in a frameshifting effect on the amino acid sequence, (4) caused loss of initiation codon, or (5) was previously reported in a confirmed, clinically affected individual with SSADH deficiency. Stop-loss variants were considered variants of uncertain significance and not included in the prevalence analysis. Variants were also excluded from prevalence estimates if determined to be benign/likely benign or if frequency in populations was greater than expected for disease. It is known that SSADHD disease is underdiagnosed and as such, is under-reported. Therefore, to better assess the status of disease prevalence and population carrier frequency, in addition to the above-described criteria for variant inclusion, *ALDH5A1* variants were further assessed using stringent CADD score prediction value of ≥28, including SpliceAI with a minimum score of >0.20 (https://cadd.gs.washington.edu). These cut-off values were determined by assessing known disease-causing pathogenic variants within these prediction programs and are inclusive of known pathogenic missense variants that are also present in gnomAD. The PHRED score of a reported pathogenic missense variant in *ALDH5A1* present in gnomAD v4.0.0 was 20.7–35, with a median CADD score of 25.7. Therefore, a high value of CADD score ≥28.0 was chosen as a best measure to ensure high stringency in selection and inclusion of potentially deleterious but unreported variants in the general population. In addition, SpliceAI score ≥0.2 was also applied, and those variants were selected to include potentially spliceogenic variants for carrier and disease prevalence estimates. This stratified assessment allowed for evaluation of disease prevalence, not only based on strictly known pathogenic/likely pathogenic variants but also on predicted deleterious variants for better estimates of the prevalence boundary.

Variants for assessment and subsequent prevalence calculations included both reported disease-associated *ALDH5A1* variants identified in individuals with documented SSADH enzyme deficiency (Supplementary Table S1), as well as pathogenic/likely pathogenic variants present in the populations represented in gnomAD (Supplementary Table S2). Carrier frequency was assessed by determining the minor allele frequency of these variants (Supplementary Tables S1, S2) across all populations present in the publicly accessible gnomAD databases. The minor allele frequency (MAF) of each variant was used to determine overall carrier frequency and disease prevalence, as described previously (Cappuccio et al., 2019; Martin et al., 2021). Disease prevalence estimates were determined based on the cumulative *ALDH5A1* pathogenic/likely pathogenic allele frequency. Data were assumed to be in Hardy-Weinberg equilibrium. Standard Hardy-Weinberg calculations were used to determine carrier frequency and disease prevalence. Our analysis estimates that the pan-ethnic prevalence of SSADHD based on reported disease-causing variants and loss-of-function variants in the general population database in gnomAD v4 is one in 564,000. However, when CADD-score based predictions of deleteriousness (CADD score ≥28.0) or splice AI (≥0.2) were applied, the pan-ethnic prevalence was estimated to be as high as one in 223,000 (Table 2).

**TABLE 2:** Estimated *ALDH5A1* carrier and SSADHD prevalence in the gnomAD v4.0.0 general population database.

Population	Carrier frequency	Disease prevalence	Carrier frequency with CADD prediction	Disease prevalence with CADD prediction	Fold-increase in prevalence
Pan-Ethnic	1/376	1/564,000	1/236	1/223,000	2.4
African/African American	1/519	1/1,000,000	1/139	1/77,000	13.0
East Asian	1/411	1/677,000	1/260	1/269,000	2.5
South Asian	1/467	1/871,000	1/261	1/271,000	3.2
Admixed American	1/375	1/562,000	1/237	1/225,000	2.5
Ashkenazi	1/2,700	1/29,500,000	1/1724	1/11,900,000	2.5
Remaining	1/308	1/377,000	1/213	1/181,000	2.1
European (non-Finnish)	1/359	1/515,000	1/238	1/226,000	2.3
European (Finnish)	1/2,292	1/2,100,000	1/553	1/1,200,000	1.8
Middle Eastern	1/428	1/732,000	1/375	1/563,000	1.3
Amish*	N/A	N/A	1/456	1/832,000	NA

* Amish alleles were not identified due to limited allele numbers in gnomAD.

Using these pathogenicity prediction estimates, among the subpopulations defined based on genetic ancestry in gnomAD v4.0.0, the African/African American subpopulation revealed the highest fold-increase in estimated disease prevalence of 13-fold when compared to estimated prevalence measured with strictly known pathogenic/likely pathogenic or truncating variants in *ALDH5A1.* Admixed American, East Asian, European (non-Finnish), European (Finnish), South Asian, and ‘remaining subpopulations’ revealed only ∼1.3- to 3.2-fold increase in estimated disease prevalence (Table 2). Amish alleles were not identified; thus, since the allele numbers are limited in gnomAD, conclusions may not be derived regarding disease prevalence in this subpopulation. Similarly, limited numbers of individuals are present in gnomAD representing both the Ashkenazi Jewish and Middle Eastern populations, which prevent firm conclusions regarding prevalence in these subpopulations.

Our analysis estimates that the pan-ethnic prevalence for SSADHD could lie somewhere between 1/223,000 to 1/564,000 based on population allele frequency data from the gnomAD v4.0.0 database (Table 2). This finding is consistent with our previously reported estimate of ∼1/460,000 worldwide (Martin et al., 2021).

Among the clinically referred population of 22,000, we identified six individuals with confirmed GABA-TD and 16 individuals with SSADHD suggesting that the disease frequency of SSADHD in this clinically referred population at our laboratory is one in 1,375 and that of GABA-TD is one in 3,500. The cumulative disease frequency of these GABA metabolism disorders in this clinically referred population at our laboratory during the time period from 2014 to 2022 is estimated to be ∼1/1,100.

### 4.3 Untargeted metabolomic analysis confirms specific biomarkers for SSADHD

Plasma untargeted metabolomic assays were obtained for a total of 13 individuals - five individuals from the BG-BCM Cohort (Cases 2, 11, 13, 14, and 15, Table 1) and eight individuals from the SSADHD Biobank (Cases 17–24; Table 1) and compared to the normal control population. These biomarker profiles were also compared to data generated from individuals with GABA transaminase deficiency (GABA-TD), a disorder that also results from altered GABA metabolism in the brain (Kennedy et al., 2019).

**FIGURE 4:**
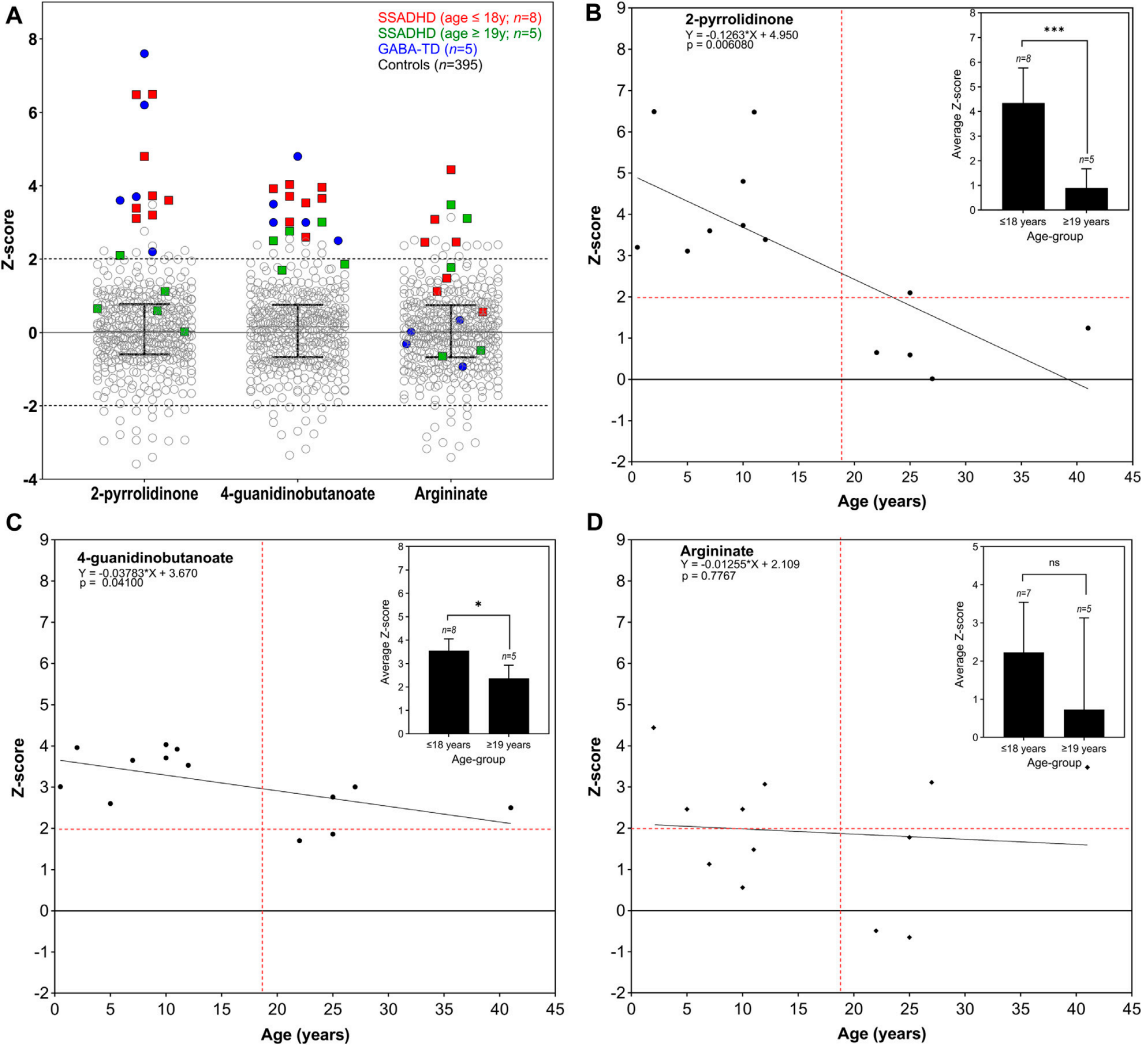
Key plasma analytes in SSADHD and GABA-TD individuals compared to control reference population. Scatter plots **(A)** are shown for plasma analyses from individuals with SSADHD and GABA-TD as compared to the control reference population (GABA-TD data shown for comparison since, as previously reported by Kennedy *et al.*, this disorder demonstrates clear defects in GABA metabolism in brain). SSADHD individuals in the pediatric age group (age ≤18 years) are indicated in red squares (*n =* 8), and those in adult age-group (age ≥19 years) are indicated in green squares (*n =* 5). Samples from GABA-TD individuals (*n =* 5) are indicated in blue circles for comparison. Control reference population (*n =* 395) is represented in open circles. Median with interquartile range is indicated for each analyte. Linear regression analysis of z-score levels by age of 2-pyrrolidinone **(B)**, 4-guanidinobutanoate **(C)**, and argininate **(D)** are shown, with difference in average z-scores of each group in inset. Dotted red lines indicate the z-score +2 on the *y*-axis and age 19 years on the *x*-axis.

Among pediatric cases (ages ≤18 years, *n =* 8), all samples (100%) showed significant elevations of both 2-pyrrolidinone (Z-score range +3.12 to +6.50) and 4-guanidinobutanoate (Z-score range +2.61 to +4.03) compared to the control cohort (*n =* 395) (Figure 4A; Supplementary Table S4). In addition, argininate, a guanidino-group containing derivative of arginine, was elevated in four of seven (57%) pediatric samples (and not reported in one pediatric sample tested on an older version of the LC-MS/MS platform). Among the adult individuals (ages ≥19 years, *n =* 5), four (80%) showed normal 2-pyrrolidinone levels (Z-score range +0.02 to +1.24), with the exception of one sample (case 22) that showed mildly elevated 2-pyrrolidinone (Z-score +2.11). 4-guanidinobutanoate was found to be high-normal or mildly elevated in all five (100%) of the adult cases (Z-score +1.70–2.38). Z-scores of both 2-pyrrolidinone and 4-guanidinobutanoate were significantly lower in the adult age group compared to the pediatric age group (*p* = 0.0004; *p* = 0.002, respectively) (Figures 4B,C). Argininate was also found to be mild to moderately elevated in plasma in 6/12 (50%) samples where this metabolite was reliably detected. Argininate was not reported in one sample (Case 2) that was assayed on a previous version of the LC-MS/MS platform. Z-scores of argininate were not significantly different between the pediatric and adult age-groups (Figure 4D).

Urine metabolomic assays were obtained from 12 individuals–four individuals from the BG-BCM cohort (Cases 11, 13, 14, and 15, Table 1) and eight individuals from the SSADHD Biobank (Cases 17–24; Table 1). In two samples (Cases 14 and 15), 2-pyrrolidinone was not reported in the LC-MS/MS platform used for the assay at the time. For the remaining 10 urine samples, 2-pyrrolidinone Z-scores were well within the normal range (Z-scores ranged from −1.005 to +0.98) (Supplementary Table S4). However, 4-guanidinobutanoate was mildly elevated in 10 of 12 samples (83%, Z-scores ranged from +1.91 to +2.78) regardless of age-group but normal in two adult samples (cases 14 and 15) (Supplementary Table S4). Argininate z-scores were normal in eight of ten (80%) samples where it was reported, and high-normal or mildly elevated in two samples (cases 17 and 18). Argininate was not reported in two samples (cases 14 and 15). Succinimide was found to be mild to moderately elevated in all pediatric urine samples (Z-score +2.09 to +3.16) and normal in all five samples in the adult age-group (Z-score +1.02 to +1.72) (Supplementary Table S4).

### 4.4 Integrated analysis of clinical, molecular, and metabolomic data allow for comprehensive diagnosis of SSADHD

Based on all available data, we have comprehensively confirmed the diagnosis of SSADHD in three individuals in BG-BCM cohort with both molecular and metabolomic analyses. Here, we present their clinical histories as a proof-of-concept of the power of this integrated diagnostic approach.

### 4.5 Case 2

This male patient was referred for testing at 6-month of age because of developmental delay and hypotonia. He was born following an uncomplicated, term pregnancy via vacuum-assisted vaginal delivery to a healthy G2P2 mother. At 2 months of age, concerns were raised due to poor weight gain. At 4.5 months of life, serum bicarbonate was measured and found to be reduced, and he was subsequently diagnosed with renal tubular acidosis and initiated on sodium bicarbonate. A dedicated renal ultrasound was normal. The patient, however, continued to have worsening hypotonia, weight loss, and lethargy. A brain MRI showed mild prominence of frontotemporal subarachnoid spaces though no structural anomalies. Urine organic acid analysis showed elevations of 4-hydroxybutyric acid, 2,4-dihydroxybutyric acid and 3,4-dihydroxybutyric acid. Subsequent exome sequencing analysis identified compound heterozygous, pathogenic variants in *ALDH5A1* (NM_001080.3:c.1015–2A>C and NM_001080.3:c.1597G>A (p.Gly533Arg) confirming the diagnosis of succinic semialdehyde dehydrogenase deficiency (Table 1). Untargeted metabolomic profiling in plasma revealed elevations of 2-pyrrolidinone and 4-guanidinobutanoate, consistent with molecular genetic findings and previous biochemical testing (Figure 4A; Supplementary Table S4).

### 4.6 Case 13

This female patient had been conceived via *in vitro* fertilization (IVF), and the pregnancy was complicated by twin gestation and the later *in utero* demise of her twin at 12–14 weeks gestation. The patient was eventually delivered at term (41 weeks) following an uncomplicated vaginal delivery. She was first noted to be hypotonic around 2 weeks of life and was notably delayed in the attainment of gross motor, fine motor, and speech milestones. At 12 months of life, her babbling decreased, and she developed a disinterest in interactive play with unusual hand postures. An MRI of the brain at the time was read as “normal”; later careful reinterpretation, however, noted subtle bilateral increased signal in the globus pallidus. The patient had normal female karyotype, *MECP2* sequencing and deletion/duplication analysis, creatine kinase (CK) level, and transferrin isoelectric focusing. Chromosomal microarray had shown only a paternally inherited deletion at 12q22.2 that was felt not to be clinically significant. Urine organic acids analysis at the age 27 months, however, demonstrated significantly elevated GHB at >500 mmol/molCr. Confirmatory Sanger sequencing at 31 months of age (2 years 7 months) revealed two pathogenic variants in *ALDH5A1* (confirmed to be in trans): NM_001080.3:c.612G>A (p.Trp204Ter) and NM_001080.3:c.1234C>T (p.Arg412Ter) consistent with her biochemical diagnosis of SSADHD (Table 1). Untargeted metabolomic profiling in plasma revealed elevations of 2-pyrrolidinone, 4-guanidinobutanoate, and argininate consistent with previous molecular genetic and biochemical testing (Figure 4A; Supplementary Table S4).

The patient has had two electroencephalograms (EEG) and has never had clinical or electrographic seizures. She has been diagnosed with perseveration/fixation, attention deficit hyperactivity disorder (ADHD), and obsessive-compulsive disorder (OCD). Given that she has never had seizures, vigabatrin has never been used in her care, though she has had a trial of taurine, lemon balm, and omega-3 DHA gel (all without significant improvement in her symptoms). She does take supplemental creatine with reported improvements in her reading, math skills, and occupational therapy goals.

### 4.7 Case 11

This patient is the younger brother of Case 13 and was 3 months old when his sister was eventually diagnosed and is now 10 years old. He was born at term (39 weeks) via an uncomplicated vaginal delivery and had a normal newborn course. Because of his siblings’ diagnosis, the patient underwent urine organic analysis at 4.5 months of life which was indeed abnormal with gross elevations in GHB (>500 mmol/molCr). He thereafter had known familial mutation testing and was confirmed to have the same two variants in *ALDH5A1* that were identified in his sister (NM_001080.3:c.612G>A (p.Trp204Ter) and NM_001080.3:c.1234C>T (p.Arg412Ter)) (Table 1). He later had a brain MRI that also showed a very subtle increase in T2 signal in the globus pallidus bilaterally with normal MR spectroscopy. Developmentally, the patient had normal muscle tone for the first 4 months of his life before becoming progressively more hypotonic beginning at the age of 6 months. He began to babble at 12 months, crawled at 13 months, and walked at 2.5 years old. He currently has about 5 words and uses sign language as his primary form of communication. He attends a school for the deaf and can sign four to 5-word sentences. Additionally, he uses a mobile device to create sentences through pictures. He has, much like his sister, never had seizures, aggression, sleep disturbances, or self-injurious behaviors, though he has been diagnosed with perseveration/fixation, attention deficit hyperactivity disorder (ADHD), and obsessive-compulsive disorder (OCD). He has never taken vigabatrin but has had a trial of taurine, lemon balm, and omega-3 DHA gel. He is currently taking creatine with reported improvements in his reading, math skills, and occupational therapy goals. Untargeted metabolomic profiling in plasma revealed elevations of 2-pyrrolidinone, 4-guanidinobutanoate, succinamic acid, and argininate consistent with previous molecular genetic and biochemical testing (Figure 4A; Supplementary Table S4).

## 5 Discussion

**FIGURE 5:**
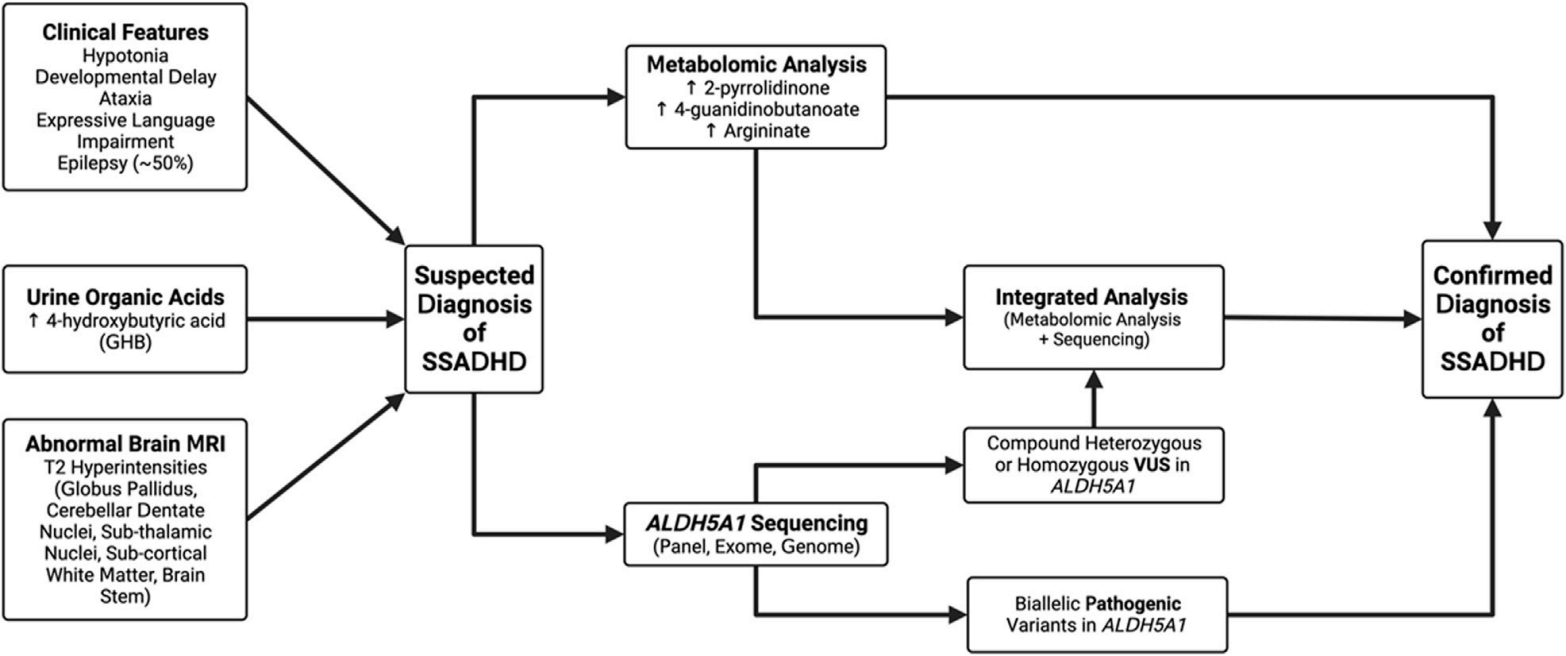
Proposed algorithm for the diagnosis of SSADHD. Patients may be suspected as having a diagnosis of SSADH deficiency based on either clinical features, abnormalities on urine organic acids, or characteristic brain imaging. Based on this, we recommend patients have clinical metabolomic analysis, genomic sequencing of ALDH5A1 or the combination of the two tests as confirmation of the diagnosis. Genomic sequencing is now considered part of the first-line approach for evaluation of a child with developmental delays.

Our study highlights the complexities inherent in the diagnosis and treatment of SSADH, as well as our attempts at mitigating some of these difficulties to improve diagnostic acumen. While this disease has now been recognized for over 30 years, there are still no disease-specific therapies available, however, in our clinically referred population the disease prevalence of SSADHD and GABA-TD disorders is estimated to be as high as one in 1000 clinical samples, which highlights the need to consider these conditions in individuals who present with neurological phenotypes. We have proposed an approach based on the combined use of minimally-invasive methods–namely, the use of untargeted metabolomic analysis in plasma and comprehensive molecular testing, in addition to a careful clinical history and readily available tests like urine organic acid analysis (Figure 5). As has been demonstrated, the singular use of either of these methods can lead to “missed” diagnoses, particularly in cases of variants of uncertain clinical significance (VUS) or in cases where the diagnosis is not clinically suspected. This approach is important because of non-specific and ambiguous phenotypes that are commonly observed in patients referred for clinical exome or genome sequencing, where VUSs confound interpretation and functional studies are required for diagnosis.

Based on our analysis of population allele frequency data, we estimate that the pan-ethnic prevalence of SSADHD may be between 1/223,000 to 1/564,000 worldwide. This determination stands in contrast to previous disease estimates and indeed confirms the suspicion that this disease is likely highly underdiagnosed in the general population, with significant underdiagnosis in less represented groups. Our study identified three novel variants in our database, and by incorporating available clinical and metabolomic data, we were able to classify the pathogenicity of these variants, thereby adding to the number of known variants in publicly available databases. While the number of identified variants was modest, we believe our approach can serve as a model for the interpretation of future VUS and hopefully, reduce the uncertainty commonly associated in the clinical sequencing results. It should be noted that in our estimation, 50% of the variants reported in individuals with SSADHD in the literature (Supplementary Table S1) are absent in the general population databases, such as gnomAD. This indicates that many disease-causing variants in SSADHD that have been reported so far are private, and their absence in the general population databases highlights the underestimation of population carrier frequency and that diverse populations are insufficiently represented in public genomic databases. Among subpopulations represented in gnomAD v4, disease prevalence was estimated to be 13-fold higher in African/African American populations when CADD and SpliceAI predictions were applied. Notably, in the African/African American subpopulation, the base prevalence was less than the pan-ethnic prevalence at one in 1,076,437; however, this estimate increased to one in 77,000 after accounting for predicted deleterious variants with CADD score (Table 2). We postulate that this difference may be an indicator of underdiagnosis and under-reporting in this subpopulation due possibly to healthcare inequities with a lack of sufficient evaluation of this population that has been well-documented (Fraiman and Wojcik, 2021). In the South Asian subpopulation, the 3.2-fold higher disease prevalence estimate may be indicative of higher disease incidence but an underdiagnosis in this population (Table 2).

Analysis of more than 900 plasma metabolites, encompassing amino acids, neurotransmitters, lipids, carbohydrates, cofactors/vitamins, purines, and pyrimidines revealed significant elevations of 2-pyrrolidinone and 4-guanidinobutanoate in all eight pediatric SSADHD individuals. 2-pyrrolidinone is a stable, lactam cyclization product of GABA, which is labile, requires a special collection protocol, and has a short half-life. We have previously shown that in patients with GABA-T deficiency, 2-pyrrolidinone and succinamic acid are significantly elevated in plasma (Kennedy et al., 2019). In SSADHD, succinamic acid was only mildly elevated in Cases 11 and 22, however (Supplementary Table S3). The elevation of 4-guanidinobutanoate is hypothesized to be due to increased conversion of GABA by glycine amidinotransferase (Figure 1B) due to accumulation of succinic semialdehyde and GABA. Unlike GABA, 4-guanidinobutanoate was not found to be an unstable compound and is reliably and consistently detected in our assay. Notably, individuals in the adult age-group had normal or high-normal plasma biomarker Z-scores. Reduction in GABA and GHB levels in patients has been reported in longitudinal studies (Johansen et al., 2017; DiBacco et al., 2019). Our cross-sectional study in individuals with SSADHD in the pediatric and adult age-groups, is consistent with previous reports. One limitation of our study is that the adult biomarker levels were compared against a pediatric reference population as they were tested in the same clinically validated metabolomic platform that is used for pediatric individuals in our laboratory. Future work may involve development of a normal adult reference cohort and reanalyze and reassess adult SSADHD individuals for a truer estimate of metabolite levels with increasing age.

Traditionally, biochemical diagnostic testing for SSADHD has required urine GHB analysis. Similarly, GABA-TD biochemical testing that measures neurotransmitter GABA levels has required a CSF sample. However, newborn screening by dried blood spot testing requires blood-based biomarkers. Therefore, identification of specific biomarkers of SSADHD and GABA-TD in plasma by metabolomic profiling not only opens an additional minimally-invasive avenue for diagnostic testing but also may make these disorders amenable to dried blood spot testing for newborn screening platforms in the future. Our analysis also identified argininate as a potentially novel biomarker for SSADHD, although not as sensitive as 2-pyrrolidinone or 4-guanidinobutanoate. Elevations in argininate, a guanidino compound and an intermediate of arginine, have not been previously reported in the context of SSADHD. Guanidino compounds have long been thought to be epileptogenic (De Deyn et al., 1991; Shiraga et al., 1991). Excess GABA is converted to 4-guanidinobutanoate via a transamidination reaction utilizing arginine (Figure 2). However, the mechanism of this elevation is unclear and requires further investigation, though this may have potential implications for our understanding of the underlying mechanism and clinical management of SSADHD. Critically, argininate may also serve as another discriminatory analyte in the development of newborn screening for SSADH should its elevations prove reliable and stable over time. Overall, metabolomic profiling of a larger cohort of patients with SSADHD will determine the specificity and sensitivity of these biomarkers and will assess utility for newborn dried blood spot analysis.

## Data Availability

The data presented in the study are deposited in ClinVar repository; accession numbers are SCV005044397, SCV005044398, SCV005044399 and SCV005044396.
